# The Role of Artificial Intelligence in Improving Patient Outcomes and Future of Healthcare Delivery in Cardiology: A Narrative Review of the Literature

**DOI:** 10.3390/healthcare12040481

**Published:** 2024-02-16

**Authors:** Dhir Gala, Haditya Behl, Mili Shah, Amgad N. Makaryus

**Affiliations:** 1 Department of Clinical Science, American University of the Caribbean School of Medicine, Cupecoy, Sint Maarten, The Netherlands; 2 Donald and Barbara Zucker School of Medicine at Hofstra/Northwell, Hofstra University, 500 Hofstra Blvd., Hempstead, NY 11549, USA; 3 Department of Cardiology, Nassau University Medical Center, Hempstead, NY 11554, USA

**Keywords:** cardiovascular diseases, artificial intelligence, cardiology, clinical decision support systems, patient outcomes, predictive models, personalized care, physician efficiency

## Abstract

Cardiovascular diseases exert a significant burden on the healthcare system worldwide. This narrative literature review discusses the role of artificial intelligence (AI) in the field of cardiology. AI has the potential to assist healthcare professionals in several ways, such as diagnosing pathologies, guiding treatments, and monitoring patients, which can lead to improved patient outcomes and a more efficient healthcare system. Moreover, clinical decision support systems in cardiology have improved significantly over the past decade. The addition of AI to these clinical decision support systems can improve patient outcomes by processing large amounts of data, identifying subtle associations, and providing a timely, evidence-based recommendation to healthcare professionals. Lastly, the application of AI allows for personalized care by utilizing predictive models and generating patient-specific treatment plans. However, there are several challenges associated with the use of AI in healthcare. The application of AI in healthcare comes with significant cost and ethical considerations. Despite these challenges, AI will be an integral part of healthcare delivery in the near future, leading to personalized patient care, improved physician efficiency, and anticipated better outcomes.

## 1. Introduction

Cardiovascular diseases are one of the leading causes of high morbidity and mortality worldwide. The disease burden has continued to increase over the past decade thereby requiring urgent interventions to prevent cardiovascular diseases and improve treatment outcomes [1]. The management of cardiovascular diseases requires a comprehensive strategy, including prevention, early diagnosis, appropriate treatment, and continuous monitoring and follow-up [2,3,4]. In recent years, noteworthy advancements in medical technology and research have significantly improved healthcare delivery in all these aspects [5,6]. However, the increasing prevalence of cardiovascular diseases, along with an aging population, highlights the urgency for implementing newer technologies to improve patient outcomes and reduce the overall burden of cardiovascular diseases.

The integration of artificial intelligence (AI) into healthcare has sparked a paradigm shift in various medical disciplines, including cardiology. AI is a field of computer science focused on creating systems or machines that can perform tasks that typically require human intelligence. It encompasses various techniques that enable machines to simulate human-like behaviors such as learning, reasoning, problem-solving, perception, and decision-making. Machine learning is a subset of AI that is a technique that allows machines to learn from vast training data without being explicitly programmed. It involves the development of algorithms that enable computers to recognize patterns within datasets, learn from these patterns, make predictions, and/or take actions based on the learned information [7,8,9]. The capabilities of AI and machine learning can be used together to revolutionize healthcare. AI’s transformative potential is evident in its applications across diagnostic imaging, personalized treatment, patient monitoring, and decision support systems. Within cardiology, AI algorithms, notably machine learning models like Convolutional Neural Networks (CNNs), have revolutionized the interpretation of medical images, such as cardiac magnetic resonance imaging (MRI), computed tomography scans (CT scans), and echocardiograms [10,11].

Natural Language Processing (NLP), another facet of AI, has recently been adopted by cardiologists to streamline the documentation process. NLP-driven solutions aid in converting spoken medical notes into comprehensive electronic health records, facilitating accurate and efficient record-keeping. Moreover, NLP algorithms contribute to data analysis within medical records, unveiling invaluable patterns and insights that aid in identifying trends and optimizing patient care pathways [12,13].

In tandem with these advancements, the integration of robotics in cardiac care holds promise for procedural precision and efficiency. Robotics-assisted interventions, such as robotically assisted surgery and catheter-based procedures, enable intricate maneuvers with enhanced accuracy, thereby reducing procedural risks and improving patient outcomes. Expert systems, a branch of AI simulating human expertise, have been applied in decision support systems within cardiology [14,15]. These systems analyze patient-specific data, leveraging accumulated knowledge and algorithms to offer tailored recommendations to healthcare practitioners. These systems, when embedded within clinical workflows, contribute to more informed decision-making and personalized patient care.

Furthermore, computer vision, an AI domain focusing on visual data interpretation, aids in the interpretation of cardiac imaging modalities, such as MRI and CT scans, enhancing diagnostic precision. Cognitive computing, an evolving AI discipline emulating human cognitive functions, augments healthcare by assimilating vast data sets, assisting in treatment planning, and predicting patient outcomes [16,17]. Affective computing, with its focus on recognizing and responding to human emotions, plays a burgeoning role in patient-centric cardiology. By analyzing patient sentiments and behaviors, affective computing contributes to personalized interventions and improved patient experiences. Reinforcement learning, an AI paradigm emphasizing decision-making through trial and error, holds the potential to optimize treatment strategies and therapeutic interventions in cardiology, fostering adaptive, patient-specific approaches [18]. Speech recognition technology, another facet of AI, facilitates seamless interaction between healthcare professionals and technology, expediting data entry processes and enabling real-time dictation of medical records [19,20]. The fusion of these diverse AI domains offers a multidimensional approach to addressing the challenges in cardiology, ranging from diagnostic precision and personalized treatments to streamlined workflows and patient-centric care.

These advancements show promise in improving diagnostic accuracy and enhancing healthcare provider efficiency. Moreover, AI has demonstrated remarkable capabilities in predicting cardiac events and personalizing treatment based on individual patient profiles [21,22]. Whether identifying early signs of cardiovascular disease, optimizing intervention procedures, or providing decision support in clinical settings, AI’s contributions to cardiology are diverse and impactful. However, the widespread integration of AI in cardiology faces challenges, including data quality, privacy concerns, regulatory frameworks, and ethical considerations. Addressing these challenges is crucial to leveraging AI’s full potential in enhancing patient care and advancing cardiology practice [23,24].

**Figure 1:**
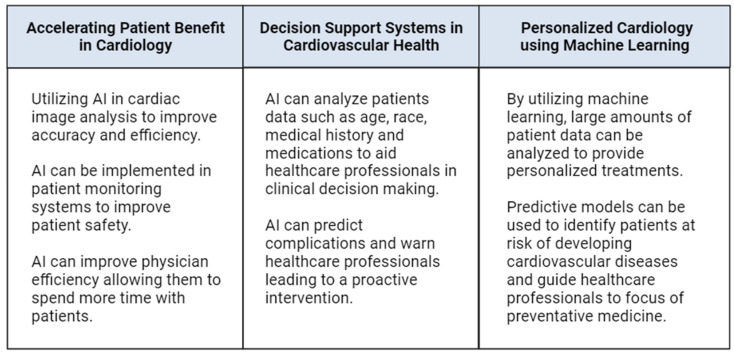
Summary of the uses of AI in the field of cardiology.

In this manuscript, we explore the role of AI in cardiology, highlighting its potential to improve diagnostic accuracy, personalize treatment, enable remote patient monitoring, and support clinical decision-making (Figure 1). Furthermore, we discuss the challenges and future directions for the responsible integration of AI technology in the field of cardiology.

## 2. Methods

**Figure 2:**
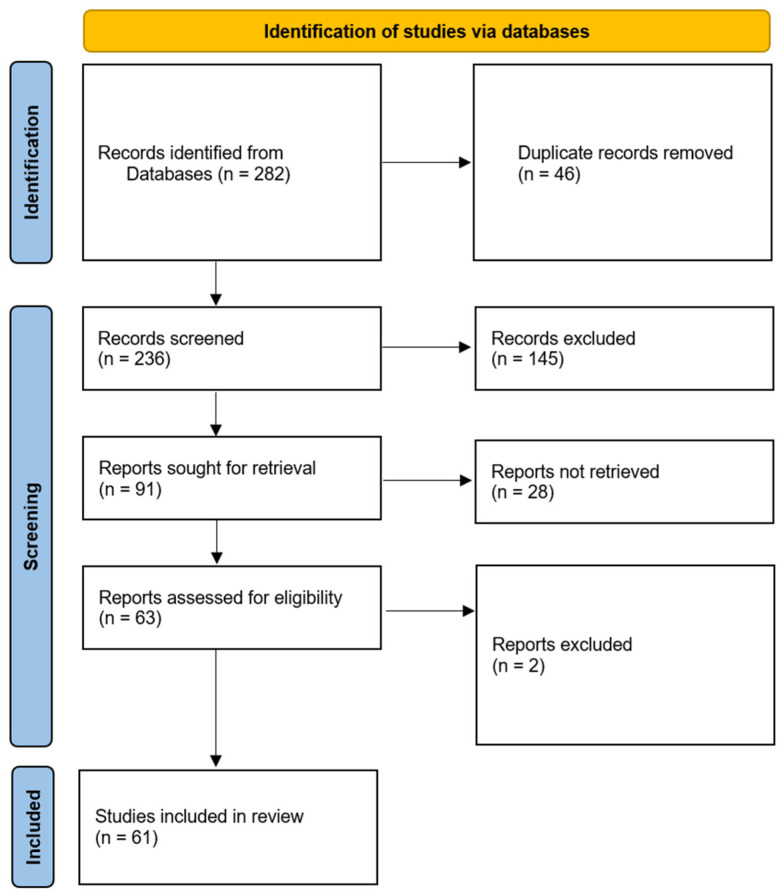
PRISMA flow diagram of study selection.

A comprehensive literature review was conducted to gather relevant articles, peer-reviewed journals, and publications related to the integration of AI in the field of cardiology. Various academic databases, including PubMed, SCOPUS, and Google Scholar, were systematically searched using keywords such as “AI in cardiology,” “machine learning,” “cardiovascular diseases,” and related terms (Figure 2). The search was limited to studies published within the last decade to ensure relevance to current advancements.

Studies and articles focusing on AI applications in cardiology, specifically in diagnostic imaging, personalized treatments, decision support systems, and patient monitoring, were meticulously reviewed. Selection criteria included relevance to AI techniques (e.g., machine learning and deep learning) in cardiology, emphasis on advancements in diagnostic accuracy, personalized care, and clinical decision support systems. The selected literature was thoroughly analyzed and synthesized to identify key themes, methodologies, and findings regarding the use of AI in cardiology. The data synthesis process involved categorizing studies based on their contributions to diagnostic accuracy enhancement, personalized treatments, decision support systems, and patient monitoring.

The quality and credibility of the included studies were evaluated based on study design, methodology, sample size, statistical analysis, and relevance to the manuscript’s focus. Only peer-reviewed studies with robust methodologies and reliable data sources were included in the synthesis. This narrative literature review employed a qualitative synthesis approach, aiming to construct a coherent narrative that synthesizes and interprets the findings from the selected studies. The narrative approach allowed for the exploration of the broader implications and potential future directions of AI integration in cardiology, beyond a mere aggregation of findings.

Limitations of the review process included the reliance on available literature, potential publication biases, and variations in methodologies among the included studies. Additionally, the rapidly evolving nature of AI technologies in healthcare may introduce limitations in capturing the latest developments.

## 3. Accelerating Patient Benefits in Cardiology Using Artificial Intelligence

AI algorithms are being used extensively within the field of cardiac image analysis [25]. Machine learning models such as CNNs have been developed and used to interpret medical images such as cardiac MRI, CT scans, and echocardiograms [26]. In a study conducted by Hannun et al. in 2019, deep neural network models outperformed cardiologists in diagnosing electrocardiogram (ECG) abnormalities and arrythmias. The deep neural networks achieved high diagnostic performance, with an average area under the receiver operating characteristic curve of 0.97 and an average F1 score of 0.837, surpassing that of average cardiologists (0.780). The results indicate that this deep learning method and AI have the potential to enhance the efficiency and accuracy of ECG analysis, decreasing the number of incorrect diagnoses in automated ECG interpretations and supporting expert-human ECG interpretation by giving priority to urgent cases. Additionally, these results show that AI has the potential to improve the accuracy of diagnosis and can be used alongside healthcare experts [27]. AI was also used in another study to predict the chances of having atrial fibrillation with the help of wearable ECG monitors. The researchers used AI and convolutional neural networks to analyze standard 10-s, 12-lead ECGs to look for the electrocardiographic signature of atrial fibrillation, even during normal sinus rhythms. They included over 180,000 patients with ECGs recorded between 1993 and 2017 and validated rhythm labels. The AI-enabled ECG has an area under the curve of 0.87, a sensitivity of 79%, a specificity of 79.5%, and an accuracy of 79.4% for detecting atrial fibrillation. This shows that AI may make it possible to identify at-risk individuals with atrial fibrillation when their sinus rhythm is normal, which may have advantages for the early diagnosis and treatment of the illness [28]. In summary, these results underscore the promising capabilities of AI and machine learning in improving diagnostic accuracy, increasing physician efficiency, and reducing errors in the field of cardiology, ultimately contributing to enhanced patient outcomes.

Another common diagnostic test in the field of cardiology is echocardiography, which can be used to diagnose various cardiovascular pathologies. To accurately diagnose cardiovascular disease, it is essential to precisely evaluate the left ventricular systolic function. Despite being real-time and non-invasive, echocardiography is subject to human error due to human experience and interobserver variability, particularly when assessing the global longitudinal strain (GLS). The use of AI and its capacity for learning to precisely identify cardiac structures, calculate ventricular volume, and assess myocardial motion has emerged as a solution. In 2021, an AI system was developed to assist in the detection of structural abnormalities within the heart, such as certain valve disorders found within echocardiographic images [29]. Furthermore, a deep-learning approach was used to automatically calculate the left ventricular ejection fractions from echocardiogram images to assess cardiac function [30]. However, while these AI applications showcase promising strides in improving echocardiography’s diagnostic accuracy, challenges persist in the widespread adoption of AI-driven solutions within clinical settings. Validation across diverse patient populations, standardization of AI algorithms, and integration into routine clinical workflows are areas of ongoing exploration and development. Furthermore, the interpretability of AI-generated assessments and ensuring seamless collaboration between AI systems and clinical expertise are pivotal for their successful implementation in enhancing echocardiography’s diagnostic capabilities. Future endeavors should focus on refining AI models to provide interpretable results, ensuring their robustness in diverse clinical scenarios and fostering synergistic collaborations between AI technology and healthcare professionals. These steps are critical in establishing AI as a reliable and indispensable tool in augmenting echocardiography for precise cardiovascular diagnoses.

In addition to echocardiography, the use of cardiac MRIs and CT scans is emerging. While no studies were found on using AI and machine learning in the field of cardiology, they have been shown to work in other medical sub-specialties. For example, the use of AI algorithms has been revolutionary in classifying and detecting diseases from MRI scans as demonstrated by a study conducted in 2018, which used AI to effectively diagnose Alzheimer’s disease from MRI images [31]. AI technology has also been effective at reading CT scans, particularly in identifying lung cancer, as seen in a 2019 study on lung cancer screening [32]. These studies suggest that there is potential to utilize AI and machine learning in the field of cardiology by recognizing pathologies on cardiac CT scans and MRIs (Table 1). By utilizing these various diagnostic modalities in conjunction with machine learning, AI can aid physicians in not only accurately diagnosing pathologies, but also allowing this to take place more efficiently. Despite these benefits, their direct implementation in cardiology requires rigorous validation, optimization, and customization to address the intricacies of cardiac imaging data and the specificity of cardiovascular pathologies. Future research endeavors should focus on tailoring AI algorithms to the nuances of cardiac MRI and CT scans, ensuring their reliability, accuracy, and seamless integration into routine clinical practice. Establishing robust AI-driven diagnostic frameworks specific to cardiology holds immense promise in revolutionizing diagnostic precision and efficiency in the field. This integration presents an exciting prospect for augmenting cardiovascular diagnostics through innovative AI-powered solutions, potentially transforming the landscape of cardiac imaging interpretation and patient care. Lastly, in scenarios where AI is employed to analyze complex medical images such as X-rays, CT scans, and MRIs, the ability to understand and interpret the explainable AI-generated results becomes crucial. Explainable AI techniques, such as saliency maps or attention mechanisms, enable healthcare professionals to comprehend the features within an image that contribute to the AI model’s decision. This interpretability fosters trust among clinicians, allowing them to validate and contextualize AI recommendations in the context of their clinical expertise.

AI has also demonstrated incredible promise in personalizing cardiovascular treatments for distinct patient profiles, including genetic variables, medical histories, and therapeutic responses [34]. Research conducted in 2019 explored the importance of personalized treatment based on individual genetic variations and its therapeutic response to the antiplatelet therapy clopidogrel [35]. Similar studies have also used AI to predict responses to statins based on machine learning and develop genetic and clinical characteristics [36]. AI demonstrates great promise within a catheterization lab by giving certain voice commands to the person operating the coronary angiography machine about how many degrees the C-arm of the machine should move. Furthermore, it can analyze the ideal angle for a patient based on various factors, such as their height and weight [33]. While performing certain procedures, such as the transcatheter aortic valve replacement (TAVR), AI can comment on the locations and sizes of the valves leading to reduced procedure times and potential complications [33]. AI can not only assist with TAVR but also with the placement of devices when it comes to other complications, such as atrial septal defect (ASD), ventricular septal defect (VSD), and patent ductus arteriosus (PDA) as well [37]. The implementation of these AI-driven systems holds immense potential to revolutionize cardiovascular care by offering tailored and precise interventions. By leveraging AI’s analytical power, these systems enable healthcare providers to deliver personalized care strategies, optimizing procedural precision, mitigating risks, and ultimately improving patient outcomes. However, the full integration of AI technologies in procedural settings requires extensive validation, refinement, and seamless incorporation into existing workflows. Future directions in this domain should focus on enhancing AI algorithms’ adaptability to diverse procedural scenarios, ensuring their accuracy, and fostering collaborative frameworks between AI technology and healthcare professionals. The successful integration of AI in procedural guidance heralds a new era in personalized cardiovascular care, potentially reshaping treatment paradigms and elevating patient care standards within cardiology.

AI plays a pivotal role in revolutionizing treatment planning and management strategies for chronic cardiac conditions, significantly enhancing personalized patient care. Through its capacity to analyze vast datasets and intricate patient profiles, AI facilitates the development of tailored treatment plans that consider individual variations in genetic predispositions, medical histories, and therapeutic responses [22,38]. By harnessing machine learning techniques, AI enables clinicians to predict treatment outcomes and tailor interventions with higher precision. Moreover, in interventional cardiology procedures like TAVR or device placements for conditions like ASD or VSD, AI assists in optimizing procedural planning, reducing complications, and enhancing patient safety. The integration of AI-driven decision support systems aids healthcare providers in delivering individualized cardiovascular care, improving treatment efficacy, and ultimately elevating patient outcomes in the management of chronic cardiac conditions [39].

Another significant benefit of integrating AI in the field of cardiology is increasing the focus on the human side of medicine. AI is a promising tool with the potential to provide invaluable support to healthcare professionals in the crucial task of medical record documentation. By utilizing AI technologies, healthcare practitioners can streamline the process of recording patient information, diagnoses, treatments, and other essential data. AI-powered solutions offer the capacity to automate data entry, categorize and code medical information, and facilitate the creation of comprehensive electronic health records [40]. Moreover, AI can assist in improving the accuracy and completeness of medical records by flagging potential errors, inconsistencies, or missing information in real time. This not only contributes to more robust patient documentation but also reduces the risk of medical errors and enhances patient safety. AI-driven natural language processing and speech recognition tools can enable healthcare providers to transcribe spoken notes into written records, further expediting the documentation process [41]. In addition to optimizing efficiency, AI can assist in data analysis and pattern recognition within medical records, aiding clinicians in identifying trends, potential risk factors, and personalized treatment options. Overall, the integration of AI into medical record documentation promises to streamline administrative tasks, enhance data accuracy, and free up valuable time for healthcare professionals to focus on providing high-quality patient-centered care [42]. While the integration of AI in medical record documentation presents significant advantages, its implementation necessitates rigorous validation and continuous refinement to ensure the utmost accuracy, reliability, and compliance with regulatory standards. Future endeavors in this realm should strive to enhance AI algorithms’ interpretative capabilities, fortify cybersecurity measures to safeguard patient data, and foster seamless integration with existing healthcare systems. The evolution of AI-driven documentation holds the potential to redefine administrative processes, laying the foundation for a more efficient, accurate, and human-centered healthcare landscape.

Additionally, AI and language models can also aid in the automation of administrative tasks in cardiology. By automating routine administrative tasks, these technologies can free up time for healthcare professionals to focus on face-to-face patient care while also improving operational efficiency [43]. Automation of these tasks can also help reduce errors or discrepancies in the patient record. The implementation of these technologies can allow healthcare professionals to spend more time with patients and bring back humanism in medicine.

There are several further benefits of integrating AI into patient monitoring systems. AI has the ability to continually assess vital signals, including heart rate, blood pressure, and respiration rate, in real-time, allowing for the early identification of deteriorating situations, earlier interventions, and improved outcomes [44]. AI calculations have been able to distinguish early indications of cardiovascular events like arrhythmias and ischemia, as shown by a study by Hannun et al. in 2019, which displayed earlier detections of atrial fibrillation from the ECG data being provided [27]. AI has already played a part in monitoring patients in all types of settings as various devices have features to monitor vital signs while providing real-time feedback [45]. A study introduced a novel algorithm combining two event-related moving averages (TERMA) and fractional Fourier transform (FrFT) for enhanced analysis of ECG signals, improving the accuracy in locating peak positions and diagnosing heart diseases. The algorithm outperformed existing methods and utilized the Shaoxing People’s Hospital (SPH) database, which included over 10,000 patients, making it a more realistic dataset for training a machine-learning model [46]. In addition to monitoring vital signs, these devices have great potential in the field of cardiology by monitoring events such as malignant arrhythmias [47]. AI can also increase the efficiency of the healthcare system by analyzing mass data quickly and accurately, leading to lower workloads on healthcare professionals, thereby enhancing patient care [48]. Lastly, wearable devices, such as smartwatches and trackers allow for remote monitoring, which provides continuous insights into the patient’s health and leads to enhanced healthcare for the patient [49]. These interventions play a pivotal role in enhancing patient safety by enabling healthcare providers to analyze extensive data efficiently and accurately, potentially leading to earlier interventions and improved prognostic outcomes. The fusion of AI into patient monitoring systems signifies a significant stride toward a more proactive and precise healthcare approach, promising a paradigm shift in patient care and safety.

## 4. Decision Support Systems in Cardiovascular Health

A decision support system in a clinical setting is designed to enhance healthcare delivery by providing targeted clinical knowledge while using specific patient data and healthcare information [50]. Traditionally, clinical decision support systems (CDSS) are mainly computerized software in which the specific patient data are then integrated with the computerized clinical knowledge to recommend the best patient-based assessments, along with recommendations that directly aid medical personnel in their clinical decision making [51]. The introduction of computers to CDSS in the 1970s was very challenging as physicians found it very time-consuming due to poor integration of the systems [52]. However, currently, the integration of electronic health records, computerized provider order entry systems, and several different web applications has led to a much more interactive and efficient CDSS [53].

The utilization of AI within the field of medical imaging has shown remarkable progress [54]. AI-learned algorithms, combined with the use of neural networks, have shown a high capacity to accurately analyze and diagnose medical images such as X-rays, CTs, and MRIs, thereby assisting clinicians with their decision making [11]. Furthermore, AI-driven CDSS provides real-time recommendations based on patient data, which include identifying any potential drug interactions, formulating specific treatment plans with the lowest side effects and risks, and predicting outcomes, which can all lead to improved healthcare. In a study, clinical decision support systems were found to significantly enhance clinical practice in 68% of cases. Univariate analyses revealed that the presence of certain system features significantly increased the likelihood of improving clinical practice. Multiple logistic regression analysis identified four independent predictors of improved clinical practice: automatic provision of decision support within clinician workflow, offering recommendations alongside assessments, providing decision support at the time and location of decision making, and utilizing computer-based decision support. Systems incorporating all four of these features demonstrated a 94% success rate in improving clinical practice [55]. In evaluating the impact of AI-driven clinical decision support systems (CDSS) and their integration into medical imaging analysis, it becomes evident that, while these systems have shown significant promise in enhancing diagnostic accuracy and aiding treatment planning, there is a need for further critical assessment. Although studies, including those indicating the success rates in improving clinical practice by integrating CDSS, have showcased substantial benefits, there remains a disparity in the comprehensive assessment of varying AI-driven CDSS models and their efficacy across different medical imaging modalities. The comparison of these findings with existing literature reveals a growing consensus on the potential of AI in medical imaging; however, the diversity of systems, data types, and clinical settings prompts a critical evaluation of the generalizability and scalability of these systems. Addressing these gaps and delving deeper into the comparative effectiveness of distinct CDSS features across diverse clinical scenarios could offer valuable insights. Moreover, future investigations might focus on refining AI algorithms to enhance interpretability and transparency, crucial for building trust and acceptance among healthcare providers, ultimately fostering seamless integration into routine clinical practice. These steps could propel the field toward more standardized, reliable, and universally applicable AI-driven CDSS in medical imaging and treatment planning.

Lastly, machine learning algorithms, when combined and integrated into electronic health records, can be used to predict patient outcomes and risks, such as myocardial infarction, sepsis, and associated complications (Table 2) [56,57]. AI stands as a transformative force in preventive cardiology through its prowess in predictive analytics. By analyzing extensive datasets encompassing patient health records, clinical parameters, and imaging data, AI models have demonstrated remarkable capabilities in risk assessment and predicting cardiac events. These predictive models leverage machine learning algorithms to identify patterns, discern risk factors, and forecast the likelihood of cardiovascular events with higher accuracy than traditional methods [58]. In a study, the performance of deep learning and machine learning models was compared to a baseline logistic regression model that used only ‘known’ risk factors to predict incident myocardial infarction from harmonized electronic health record data. The research involved a large-scale case-control study with data from 2.27 million patients, including 20,591 patients diagnosed with MI. While a deep neural network with random under-sampling showed the best classification performance, it provided only a moderate improvement over traditional methods. The F1 Score was 0.092, and the area under the curve was 0.835, suggesting that deep neural networks may not offer a significant advantage when trained on harmonized data compared to using well-established risk factors for MI in logistic regression models. The calibration of all models was suboptimal due to overfitting related to the low frequency of MI cases [57]. Machine learning and deep learning applications for predicting sepsis using electronic health records have gained attention for early intervention. A systematic review of 42 selected studies, out of 1942 articles, revealed the predominance of retrospective studies, varying data sources, and sepsis definitions and the importance of data augmentation. The review highlighted the potential of ML/DL methods in sepsis detection and early prediction using electronic health records data [56]. This led to a more proactive intervention by the healthcare team. While these approaches show promise in early detection using electronic health records, the predominance of retrospective studies and variations in data sources underscore the need for standardized data practices and consistent definitions for robust predictive models. These findings prompt a call for further research focusing on refining model calibration, enhancing data quality, and establishing standardized protocols, ensuring the reliability and scalability of ML-based predictive analytics in preventive cardiology. This could propel these technologies toward more effective clinical applications and proactive healthcare interventions.

In the landscape of AI cardiology, the algorithms employed play a pivotal role in shaping CDSS. Traditional CDSS often relies on rule-based systems, where expert knowledge is encoded into explicit decision rules. However, the integration of machine learning algorithms has brought about transformative changes. Supervised learning algorithms, such as Support Vector Machines and Random Forests, leverage historical patient data for prediction tasks, while unsupervised learning algorithms, including clustering methods, contribute to identifying patient subgroups and disease patterns [59,60]. In particular, in medical imaging, convolutional neural networks (CNNs) have demonstrated significant promise in enhancing diagnostic accuracy, especially in interpreting cardiovascular imaging. As the field progresses, the need for transparency and interpretability in AI-driven decision making becomes increasingly important. Explainable AI algorithms, integrated into CDSS, offer a way to demystify complex model outputs. Decision trees, with their clear decision paths and model-agnostic techniques like LIME, provide interpretable explanations for individual predictions. This emphasis on explainability not only fosters trust among healthcare professionals but also ensures that AI recommendations align with the logic of clinical expertise [61,62].

Explainable AI addresses a fundamental challenge associated with conventional AI models, particularly deep learning algorithms, which are often considered “black boxes” due to their complexity in making predictions. In the context of CDSS, where accurate and trustworthy decision support is paramount, the interpretability of AI models becomes critical. Explainable AI methods aim to provide transparency in AI-driven decision making, offering insights into how algorithms arrive at specific conclusions [61]. This transparency is especially relevant when these systems are assisting healthcare professionals in critical decisions regarding patient care.

The implementation of AI-guided CDSS has many benefits and challenges. The data-driven insights and support provided by CDSS can lead to better decision making and enhanced decision quality [63]. The use of CDSS has led to significantly reduced decision-making time, leading to earlier interventions and enhanced efficiency in terms of the healthcare provided [64]. The integration of data with the use of computer software and CDSS has provided medical personnel with a more comprehensive view of healthcare in general [65]. Many CDSSs help with personalization and patient-tailored care as they are user-specific [66]. However, CDSS depends on the quality of the data provided, and any significant issues regarding data quality can lead to skewed results [67]. Furthermore, the implementation and changes of CDSS can be challenging as certain integration, user training, and infrastructures are often required for such modifications [68]. Lastly, the high initial and ongoing expenses of CDSS systems prevent their widespread use and is currently an ongoing challenge, which requires careful cost-benefit analysis [69].

## 5. Personalized Cardiology Using Machine Learning

Personalization in cardiac care is a developing topic that tries to customize medical interventions and treatments specific to patients based on their needs. A study conducted in 2015 used personalized genetic testing and risk assessments to identify the underlying pathophysiology of complex cardiovascular disorders and thereby allowed for more focused treatment and follow-ups [70]. Currently, there are personalized treatments for conditions such as heart failure, hypertension, and myocardial infarction, and there is an overall increased focus on multiple factors that allow for a tailored treatment based on individuals’ particular conditions [71]. Personalized care extends beyond pharmacotherapy or surgery and can include lifestyle modifications focused on assisting individuals efficiently [72].

Machine learning techniques have been crucial in personalized medicine within the field of cardiology. Machine learning was accurately used to identify and predict high-risk patients with ST-elevation myocardial infarction (STEMI), highlighting the importance of machine learning in preparing risk assessments and personalized plans for patients [73,74]. A study conducted in 2017 highlighted a deep learning model that consisted of cardiologist-level accuracy in identifying and diagnosing arrhythmias, leading to much-needed interventions and monitoring within patients. This study focused on automating the evaluation of strain in two populations of patients with acute myocardial infarction. Automated and manual analyses were compared for agreement and predictive value in major adverse cardiac events (MACE). The automated analysis showed good agreement with manual assessment for GLS and global circumferential strain (GCS), and GLS was the only independent predictor of MACE among the automated analyses, indicating the potential for automation to enhance efficiency and clinical implementation [75]. Along with identifying and diagnosing conditions, machine learning has been able to analyze data to offer remote monitoring and telemedicine catered toward individual patients. Previous research has identified rhythm abnormalities such as atrial fibrillation with the use of personal devices that have AI-enabled algorithms, allowing for timely and personalized interventions. A novel algorithm for detecting sudden cardiac arrests on ECG aimed at improving the performance of automated external defibrillators. The algorithm combined a convolutional neural network as a feature extractor and a Boosting (BS) classifier, demonstrating high accuracy in sudden cardiac arrest detection. By employing a grid search and nested five-fold cross-validation, the CNNE was trained on preprocessed ECG data, leading to impressive results with an accuracy of 99.26%, sensitivity of 97.07%, and specificity of 99.44%. This approach enhanced the performance of the shock advice algorithm (SAA) for AEDs in identifying shockable rhythms, such as ventricular fibrillation and ventricular tachycardia [28,76]. The studies showcasing machine learning’s prowess in cardiology present promising advancements in diagnosis, risk prediction, and remote monitoring. However, it is crucial to critically assess these findings by considering their clinical applicability and limitations. While ML algorithms exhibited impressive accuracy in identifying high-risk patients for conditions like STEMI and predicting MACE, the translation of these findings into real-world clinical settings warrants cautious consideration. The generalizability of these models across diverse patient populations, the scalability of implementation in healthcare systems, and the potential biases inherent in training data need careful examination. The high accuracy rates reported in these studies raise optimism, but a critical view must consider the potential impact on real-time clinical decision-making, patient outcomes, and the extent to which these algorithms might contribute to improving healthcare workflows and efficiency. These critical analyses help contextualize the advancements made by ML models in cardiology and emphasize the need for comprehensive validation and real-world testing to ensure their clinical effectiveness and widespread applicability.

Machine learning techniques have also been able to use cardiac CT scans, along with the coronary artery calcium score (Ca Score) framework, to automatically evaluate coronary artery calcium scores, which have then allowed for more personalized assessments of cardiovascular risk [77,78]. The first study aimed to evaluate methods for coronary artery calcification scoring in cardiac CT, which is an essential predictor of cardiovascular disease events. Four cardiovascular disease risk categories and data from 72 patients, including calcium scoring CT (CSCT) and coronary CT angiography (CCTA) scans, were utilized. The (semi)automatic approaches diagnosed 52% to 94% of coronary artery calcification lesions correctly, with positive predictive values ranging from 65% to 96%. These methods demonstrated the feasibility of automatically categorizing patients’ cardiovascular disease risk despite challenges in detecting coronary artery calcification lesions at certain locations. Overall, this standardized framework facilitates coronary artery calcification scoring assessment in cardiac CT [77]. Additionally, another study looked at an automated method for quantifying coronary artery calcification in cardiac CT angiography (CCTA) scans, which is a strong predictor of cardiovascular events. This method utilizes supervised learning and does not require coronary artery extraction. A bounding box around the heart is determined automatically, and convolutional neural networks (ConvNets) are used to identify and quantify coronary artery calcification. An ensemble of ConvPairs achieved a sensitivity of 71%, with 0.48 false positive errors per scan, demonstrating high accuracy and excellent agreement with reference annotations in CCTA and CSCT. This method may eliminate the need for a dedicated calcium scoring CT scan and reduce radiation dose in patient care [78]. While the (semi)automatic methods showcased commendable accuracy rates in detecting coronary artery calcification lesions, the variability in performance across different cardiovascular disease risk categories and lesion locations warrants careful consideration. The observed challenges in detecting lesions at specific sites underscore potential limitations in the algorithm’s generalizability to diverse patient populations and the need for further refinement. Future research endeavors should focus on validating these automated methods across broader patient cohorts and diverse imaging protocols to ensure their reliability and accuracy across varied clinical scenarios. Exploring the integration of these algorithms into routine clinical workflows and assessing their impact on cardiovascular risk assessment and patient outcomes will be crucial for their effective implementation in clinical practice (Table 3).

Clinical predictive models aid healthcare professionals and patients by assessing and informing them about risks in terms of their health, with the aim of assisting decision making and leading to improved outcomes [79]. A widely used predictive model for determining the risks of developing CAD over a 10-year period is the Framingham risk score (FRS) [80]. The FRS considers factors such as total cholesterol, HDL, age, gender, smoking, and systolic blood pressure, which then assist in determining the risk of CVD and providing patient-specific modifications in order to lower risks [81]. With the developments of machine learning and artificial intelligence, various models have been used to analyze clinical and imaging data to interpret and generate risk prediction. Based on these risk predictions, specific care and aid have been provided to patients, leading to better outcomes [17]. A study used machine learning for the prediction of all-cause mortality in patients suspected of CAD and helped clinicians with patient-specific care based on their informed decisions and the risk stratifications provided by the predictive model [82]. Overall, the field of predictive modeling for patient-specific care holds great promise, leading to more patient-centered and data-driven care, progressing to improved healthcare quality in the coming decades [83]. Future research should focus on validating these ML-driven predictive models across larger and more diverse patient cohorts to ascertain their reliability, generalizability, and clinical utility. Furthermore, assessing the long-term impact of these models on healthcare quality and patient outcomes will be crucial for their effective integration into routine clinical practice. Collaborative efforts between researchers, healthcare providers, and data scientists are imperative to harness the full potential of predictive modeling in advancing patient-centered care and improving healthcare quality in the years ahead. Lastly, as machine learning techniques are leveraged to predict outcomes, recommend therapies, and identify high-risk patients, understanding the rationale behind these predictions becomes paramount [84]. Explainable AI methods offer a means to elucidate the decision-making process of complex models, providing healthcare professionals with insights into the features and patterns contributing to personalized recommendations.

## 6. Challenges and Future Directions

The increasing use of AI in the field of cardiology presents both exciting opportunities and significant challenges. Since AI produces outputs based solely on its training data, one of the challenges is obtaining high-quality data for AI training and validation. Not only can it be challenging to gather datasets for rare heart conditions, but also for patient populations that are not well represented [5,85,86]. Moreover, these training datasets that will be used for training need to be blinded or masked appropriately to ensure patient privacy. These tools will require high security measures to prevent breaches or leaking of any data. Additionally, AI systems have a tendency to replicate any bias that may be present in their training dataset [87,88].

There are several ethical considerations that need to be made when utilizing AI in healthcare. Securing sensitive patient data for training AI systems is not only a collection challenge but also a vital element in preserving patient privacy. Robust encryption, access controls, and regular monitoring are necessary to protect data from unauthorized access and breaches. Incorporating privacy-preserving techniques like federated learning can reduce data exposure risks while ensuring the security of training data. Overall, maintaining data security is paramount for healthcare AI, ensuring the highest standards of patient privacy and data protection. In addition, there may be concerns about liability and malpractice because AI systems can potentially lead to a misdiagnosis or mistreatment, leading to patient harm [89,90].

Cost and implementation present substantial hurdles in the widespread integration of AI technology within cardiology. The initial investment required for adopting AI-driven solutions, including the procurement of advanced hardware, software development, and staff training, poses a significant financial burden on healthcare systems [91,92,93]. Moreover, the ongoing maintenance and updates demand continuous investment. These expenses might limit accessibility and adoption, particularly in healthcare settings with constrained resources. The integration process itself demands a comprehensive restructuring of existing workflows and infrastructure, which can be complex and time-consuming. Furthermore, interoperability issues with existing systems and the need for seamless integration into clinical practice add layers of complexity to the implementation process. Addressing these challenges requires not only financial investment but also strategic planning, standardized protocols, and collaboration among stakeholders to ensure a smooth and cost-effective integration of AI technologies into cardiology practice.

Another notable concern revolves around the potential dependency on AI technology within cardiology practice. While AI presents promising capabilities, over-reliance on automated systems without adequate validation or human oversight could introduce risks. Being entirely dependent on AI-generated insights or recommendations might lead to complacency or errors in case of technological failures, inaccuracies, or unforeseen circumstances [94,95]. It is imperative to maintain a balanced approach, integrating AI as a supportive tool rather than a replacement for clinical judgment and expertise. Establishing protocols for continuous validation, human supervision, and thorough verification of AI-driven outcomes becomes paramount. Moreover, fostering a culture that encourages critical appraisal of AI-generated outputs and encourages collaboration between healthcare professionals and technology can mitigate the potential risks associated with overreliance on AI in cardiology. Lastly, While AI algorithms excel in analyzing structured medical data, their ability to account for external factors influencing health, such as interpersonal ties and social determinants, presents inherent challenges. AI predominantly relies on available healthcare data, often focusing on clinical parameters and biomedical markers. Incorporating external socio-environmental factors, including interpersonal ties, presents complexities due to the lack of standardized data collection and integration [43,96,97].

Despite these challenges, the future of AI in the field of cardiology is promising. Establishing clear guidelines and robust security measures for the utilization of patient data in AI system training is imperative. These safeguards are essential to protect patient privacy, maintain data integrity, and uphold ethical standards in healthcare AI development. Such guidelines should encompass strict data access controls, encryption protocols, and compliance with data protection regulations. Security measures should include data anonymization or pseudonymization to prevent the identification of individual patients. Access to patient data should be restricted to authorized personnel only, and audits should be conducted to monitor data usage. Furthermore, consent and transparency are vital components of using patient data for AI training. Patients should be informed about how their data will be used and have the opportunity to provide or withhold consent. Guidelines should also address the sharing and storage of data, ensuring that data remain secure throughout its lifecycle [98].

In the rapidly evolving landscape of AI in healthcare, stringent regulation and vigilant monitoring play a pivotal role in upholding patient safety. The application of AI technology has immense potential to revolutionize healthcare, but it also introduces unique challenges, particularly regarding patient data privacy, system validation, transparency, accountability, and ethical considerations. Close regulatory oversight ensures that AI systems adhere to the highest standards of performance, data security, and ethical practice. By striking the right balance between innovation and safety, we can harness AI’s capabilities to enhance patient care while safeguarding the well-being and privacy of individuals within the healthcare ecosystem. Lastly, continuous education and training are of paramount importance to ensure that healthcare professionals can effectively and consistently integrate AI systems into their clinical practice. As AI technologies evolve and become more prominent in healthcare, it is essential for medical practitioners to stay updated with the latest developments. Continuous education programs can empower healthcare professionals with the knowledge and skills needed to harness AI’s full potential, enabling them to make informed decisions, interpret AI-generated insights, and seamlessly incorporate AI tools into their daily routines. This ongoing training not only enhances patient care but also ensures that AI is used in a responsible and ethical manner, aligning with the evolving landscape of healthcare delivery [99].

## 7. Conclusions

In conclusion, this narrative literature review highlights the potential of AI technology to improve the quality of care delivered to patients. Through different applications, such as cardiac imaging analysis, individualized therapy recommendations, real-time patient monitoring, and decision support systems, AI-driven algorithms have shown immense promise. AI has the potential to improve cardiovascular disease prevention, diagnosis, treatment, and monitoring, thereby improving patient outcomes. Despite these beneficial aspects, the application of AI in cardiology has a few concerns to overcome, such as data quality and privacy. In addition, AI technology used in cardiology needs to be well-regulated and monitored. AI has the potential to greatly lower the burden of cardiovascular diseases and improve the overall quality of cardiac treatment for patients worldwide if approached and implemented correctly.

## 8. Key Points

AI enhances diagnostic accuracy in interpreting various cardiac images, such as CT scans, MRIs, and echocardiograms.

AI and language models streamline medical record documentation, automate administrative tasks, and improve operational efficiency, enabling healthcare professionals to focus more on direct patient care.

Wearable devices and AI-powered systems facilitate remote patient monitoring, allowing continuous real-time assessment of vital signs, early detection of cardiovascular events, and a proactive healthcare approach.

AI enhances clinical decision support systems, improving diagnostic accuracy and aiding treatment planning in cardiovascular health.

Personalized cardiology benefits from machine learning, providing tailored interventions, high-risk patient identification, and patient-specific treatments.

Challenges in AI implementation include obtaining quality data, addressing biases, ensuring data privacy, and addressing ethical concerns and liabilities.

Future directions emphasize refining AI algorithms, validating predictive models, and implementing guidelines for responsible AI use, with continuous education for healthcare professionals.

## Figures and Tables

**Table 1:** Overview of AI applications in cardiology studies, highlighting diagnostic modalities, performance metrics, and future considerations.

Study	AI Application	Diagnostic Modality	Performance Metrics	Future Considerations
[27]	Deep neural networks for ECG analysis	ECG	AUC: 0.97 and F1 score: 0.837	Interpretability and collaboration between AI systems and clinical expertise
[28]	AI-enabled ECG for atrial fibrillation prediction	Wearable ECG monitors	AUC: 0.87, sensitivity: 79%, specificity: 79.5%, and accuracy: 79.4%	Considerations for undetected atrial fibrillation and prospective calibration before widespread application to a broader population
[33]	AI in coronary angiography and TAVR	Coronary angiography and TAVR	Procedure time and complication rate reduction	Enhancing AI algorithms’ adaptability to diverse procedural scenarios

**Table 2:** Overview of AI applications in CDSS for cardiology, detailing their implementation in clinical settings, performance metrics, and future considerations.

Study	AI Application	CDSS in Clinical Settings	Performance Metrics	Future Considerations
[55]	AI-driven CDSS in clinical practice	Real-time recommendations based on patient data	68% improvement in clinical practice	Disparity in assessing various AI-driven CDSS models
[56]	AI-driven CDSS for sepsis prediction	Predicting sepsis outcomes	Potential in early sepsis detection	Challenges in EHR data quality and standardization Prospective validation studies for clinical impact assessment.
[57]	AI-driven CDSS for myocardial infarction prediction	Predicting myocardial infarction outcomes	Moderate improvement over traditional methods; F1 Score: 0.092 and AUC: 0.835	Calibration challenges due to overfitting from low-event frequency Adequate discrimination despite poor calibration

**Table 3:** Overview of machine learning applications in various cardiology-focused areas, highlighting performance metrics and future considerations.

Study	Focus Area	Machine Learning Application	Performance Metrics	Future Considerations
[73]	Risk prediction in resource-limited countries	STEMI	Improved mortality prediction following STEMI Extra Tree ML model demonstrated best predictive ability (sensitivity: 85%, AUC: 79.7%, and accuracy: 75%)	Clinical applicability Generalizability across diverse patient populations Reducing biases in training data
[75]	Automated volume-derived cardiac functional evaluation	CMR imaging and automated strain assessment	GLS and GCS best predicted MACE with high accuracy	Time-consuming post-processing Validation in broader populations
[77]	(Semi)Automatic CAC identification in cardiac CT	Cardiac CT and automated CAC scoring	1. Detection of 52% to 94% of CAC lesions. Positive predictive values between 65% and 96%. 2. Linearly weighted Cohen’s kappa for patient CVD risk categorization ranged from 0.80 to 1.00.	Missed lesions in distal coronary arteries False positive errors near coronary ostia Challenges in ambiguous locations

## Data Availability

Data are contained within the article.
